# Supplementary material for: Chromothripsis during telomere crisis is independent of NHEJ, and consistent with a replicative origin
Source: Genome Res. 2019 May;29(5):737–49. doi: 10.1101/gr.240705.118 (PMC6499312; doi:10.1101/gr.240705.118)
Supplement: Supplemental Material [file supp_gr.240705.118_Supplemental_file_1.zip › contigs/annotated_contigs/DB78/contig.2.DB78_length_679_mean_cov_13.1001472754.docx]

**DB78_length_679_mean_cov_13.1001472754**

```
GGCATACGAGATTCTCCGGAGTGACTGGAGTTCAGACGTGTGCTCTTCCGATC|TCATGCTTTGCTTTAAAAAATAAATAAATAAATGC
                                                      >chr6:159177424-159177749 + E=9e-1
AGGCTGGTGCAGTGGCTCACGCCTGTAATCCCAGCAATTTGGGAGGCCGAGGCAGGTGGGTCATTTGAGGTCAGGAGTTCGAGACCAAC
84
CTGGCCAACGTGGTGAAACCCTGTCTCTACTAAAAATACAAAAAAACAAGCCGGGCGTGGTGTTGGGCGTCTATAGTCCCAGCTACTCA

GGAGGCTAAGGCAGGAGAATCGCTTGAACCCAGGAGGCAGAGGTTGCAGTGAGCCAAGATCGTGCCACTGCACTCCAGCCTGGTGACAG

AGCGAGATCCTGTCTCAAAAAAA|TTTT|TTCATAGAGAAAGATAGCATATTTTGAAAAACGTGCAGATTGGCATATATTTGTGATTTT
                            >chr6:159101941-159102238 + E=6e-167
ACTTACATTAGCAAGCATTAATTTTCTAAAAAATCCACTTGAGTTCATGTATAGCCTGAAAGAACCTAATGGAGAATTTATTTCAGTCA

ATACTGTAATTTATAGAAGAGAATCAGTCATGAGTTGAAGTTTTAATTTTTTTCTTAAATTTGCATAACAAAGGAATTGGCCAGTTTTG

TGGCACATTATTTTAAGGATGTGAAGATCTTATAATATTTACATGTTTGCCTTTTTATA
```
